# Ventilator-associated pneumonia in critically-ill patients with COVID-19 in a setting of selective decontamination of the digestive tract

**DOI:** 10.1186/s13054-021-03869-y

**Published:** 2021-12-20

**Authors:** Sinta B. van der Meer, Grace Figaroa, Peter H. J. van der Voort, Maarten W. Nijsten, Janesh Pillay

**Affiliations:** 1 Department of Critical Care, University Medical Center Groningen, University of Groningen, Hanzeplein 1, 9713 GZ Groningen, The Netherlands; 2 Department of Pathology and Medical Biology, Groningen Research Institute for Asthma and COPD, University Medical Center Groningen, University of Groningen, Groningen, The Netherlands

**To the editor,**

In mechanically ventilated patients with COVID-19 high incidences of ventilator-associated pneumonia (VAP) have been reported ranging from 40 to 58% [1, 2]. This occurred despite judicious use of systemic antibiotics at ICU-admission, preventive measures and in healthcare systems with more than adequate staffing resources [3].

In the Netherlands, most patients admitted to an ICU receive a regimen of selective decontamination of the digestive tract (SDD), aimed at eradication of pathogenic flora and preservation of protective anaerobic bacteria [4]. This consists of a 3rd generation cephalosporin for 4 days intravenously, topical and enteral nonabsorbable antibiotics (polymyxin, tobramycin, amphotericin B) during their entire ICU stay [4]. SDD has reduced nosocomial infections and mortality in patient populations with an overall shorter duration of mechanical ventilation and ICU stay (8–9 days) compared to the current patients infected with SARS-CoV-2 [5].

We assessed our practice of care, including SDD, and the associated incidence of VAP in patients infected with SARS-COV-2 and compared it to current literature. We performed a single center retrospective observational study in the University Medical Center of Groningen (UMCG), The Netherlands. All adult patients consecutively admitted to our ICU between March 2020 and February 2021 with PCR-confirmed COVID-19 were included. This resulted in inclusion of 212 mechanically ventilated patients. Standard care with SDD included microbiological surveillance of respiratory samples, throat and rectal swabs at admission, and twice weekly thereafter. All patients were retrospectively reviewed for presence of VAP. VAP was defined accordingly by the presence of clinical suspicion (fever and/or decline in ventilation or oxygenation), laboratory parameters (leukocyte count and CRP), new or progressive radiographic infiltrates and positive microbiological cultures from lower respiratory tract specimens (surveillance and obtained additionally when VAP was suspected).

Twenty-two patients (10%) had confirmed VAP and the median time to diagnosis was 12 (IQR 7–17) days (Table 1). The observed low VAP incidence of 10%, occurred despite the fact that 96% of the patients were mechanically ventilated for more than 5 days. This incidence is in contrast with aforementioned high rates of 40–58% [1]. 62% of patients received steroids during ICU admission, the percentage of VAP was not higher compared to patients not receiving steroids (11% vs 9.6%).

**Table 1:** Characteristics of patients with and without VAP

	No VAP *n* = 190 (90%)	VAP^a^ *n* = 22 (10%)	*p* value
Age	63 (56–70)	65 (54–23)	.75
Gender (female)	57 (30%)	4 (18%)	.32
BMI > 30	78 (41%)	7 (32%)	.49
Diabetes mellitus	54 (28%)	4 (18%)	.45
Hypertension	75 (40%)	9 (41%)	1.00
Chronic kidney disease	16 (8%)	0	.38
Chronic lung disease	25 (13%)	6 (27%)	.10
Immune compromised	24 (13%)	0	.14
SOFA-score	6 (4–7)	7 (4–7)	.26
Time to VAP (days)	na	12 (7–17)	
Use of SDD	189 (99.5%)	22 (100%)	1.00
Corticosteroids	118 (62%)	15 (68%)	.65
ECMO	12 (6%)	3 (14%)	.19
CRRT	24 (13%)	2 (9%)	1.00
Proning during MV	107 (56%)	19 (86%)	.006
Length of MV (days)	13 (8–21)	26 (15–33)	< 0.0001
Length of ICU stay (days)	15 (9–22)	25 (21–35)	< 0.0001
ICU mortality	57 (30%)	9 (41%)	.33

Data are reported as median (IQR-range) or *n* (%). *p* values were calculated using Mann–Whitney *U* test and Chi-Square test in SPSS

^a^Positive cultures contained *S. aureus* (*n* = 7), *P. aeruginosa* *n* = 4), *S. marcescens* (*n* = 3), *S. paucimobilis* (*n* = 2), *K. pneumoniae* (*n* = 2), *E. coli*, *P. agglomerans*, *A. fumigatus* and *Proteus mirabilis*. Low pathogenic bacteria (enterococci and bacillus) were excluded from our VAP definition

We recognize the limitations and risk of bias and underdiagnosis when retrospectively identifying VAP, however there was a 90% agreement between clinically (by the treating intensivist) and retrospectively identified VAP. The observed low incidence of VAP could be attributed to underdiagnosis, although routine microbiological surveillance would likely have resulted in an overestimation of VAP. Additionally, judicious use of antibiotics could have artificially reduced rate of VAP using microbiological confirmation, however apart from the 4-day course of cephalosporins, use of antibiotics was limited (44% of all patients after hospital admission) compared to rates > 80% reported elsewhere[2].

The main limitation of this single center observational study is the lack of a control group receiving no SDD, therefore a causal relationship between the use of SDD and the incidence of VAP cannot be established. Demographics, treatments and outcomes between patients described in this report and previous literature are similar, however major differences are the use of SDD and the incidence of VAP [1–3]. In support of our data, a recently published observational study, suggests a strong mortality benefit of SDD, although the incidence of VAP was not reported [6].

Therefore, although no causal relationship can be established from this report, our practice of care including the use of SDD appears to be associated with a reduced incidence of VAP in critically-ill patients infected with SARS-COV-2 as reported in other critically-ill patients [5].

## Data Availability

Not applicable.
